# COVID-19 and functional disability: current insights and rehabilitation strategies

**DOI:** 10.1136/postgradmedj-2020-138227

**Published:** 2020-08-04

**Authors:** Pasquale Ambrosino, Antimo Papa, Mauro Maniscalco, Matteo Nicola Dario Di Minno

**Affiliations:** Istituti Clinici Scientifici Maugeri IRCCS, Pavia, Italy; Istituti Clinici Scientifici Maugeri IRCCS, Pavia, Italy; Istituti Clinici Scientifici Maugeri IRCCS, Pavia, Italy; Department of Translational Medical Sciences, “Federico II” University, Naples, Italy

## RESEARCH LETTER

In December 2019, the novel severe acute respiratory syndrome coronavirus 2 (SARS-CoV-2) made the first appearance in Wuhan, China. Despite the attempts to minimise its spread, SARS-CoV-2 gave rise to a public health emergency, finally resulting in the WHO declaration of pandemic in March 2020.

Epidemiologic data indicate that SARS-CoV-2 causes the coronavirus disease 2019 (COVID-19), a condition with a wide spectrum of clinical presentations, ranging from a mild disease with influenza-like symptoms to a severe form with acute respiratory distress syndrome (ARDS), requiring specialised management at intensive care units (ICU).

The long-term consequences of surviving the most severe form of SARS-CoV-2 infection are still largely unknown. However, based on the scientific evidence currently available, some preliminary considerations can be made on this issue.

To date, spirometry indicates a good capacity of recovery in terms of lung function after ARDS. The Toronto ARDS Outcomes Study Group investigated the computed tomography (CT) scans of patients with ARDS at 5-year follow-up, showing a complete resolution of consolidation in all survivors and less than 10% of patients with mild residual CT abnormalities.^1^

If the lung seems to be an organ with a good capacity of functional recovery, a series of residual limitations in terms of exercise capacity and quality of life have been observed following ARDS and ICU stay, with significant impact on the costs and the need of healthcare services.

Regardless of the underlying pathology, the post-intensive care syndrome (PICS) is a well-known condition, characterised by residual physical and cognitive limitations in ICU survivors. The main clinical manifestations of PICS include fatigue, weakness and exercise intolerance, with different degrees of sexual, sleep, mood and cognitive disorders. As a consequence, family members can be physically and psychologically affected both during ICU stay and after discharge, thus resulting in the PICS-Family (PICS-F). During SARS-CoV-2 pandemic, it is important to consider that PICS and PICS-F may significantly influence post-discharge costs and utilisation of healthcare services.

In a cohort of 109 patients with ARDS, the Canadian Critical Care Trials Group documented persistent limitations in terms of quality of life and exercise capacity among survivors 5 years after discharge from ICU.^2^ In detail, as compared to a healthy control population, the authors reported a reduced distance at the 6-minute-walking-test (6MWT), which was consistent with a concomitant reduction in the physical component of the 36-Item Short Form Health Survey. In addition, several physical and psychological problems affected family caregivers. Overall, because of the residual functional disability, the cumulative post-discharge healthcare costs per patient per year (from $5,000 CAN to $6,000 CAN) were comparable to those of elderly patients with severe chronic diseases.

The observation that young healthy subjects without important comorbidities do not return to a baseline functional status and to baseline levels of healthcare necessity after ARDS and ICU stay may have important public health implications, particularly in the frame of the current global emergency due to SARS-CoV-2 pandemic.

Most clinical studies have shown that myalgia and fatigue are frequent symptoms in patients with COVID-19, regardless of the severity of the disease. It has been hypothesised that these symptoms are a consequence of the interaction between concomitant chronic diseases and the acute inflammatory state of the systemic SARS-CoV-2 infection. Moreover, in critically ill patients, the prolonged sedation and immobility in the ICU may play a key role in the genesis of the so-called ICU-acquired weakness (ICU-AW), which is a state of chronic fatigue that compromises the patient’s physical abilities and his mental health for months or years after discharge. Critical illness myopathy and polyneuropathy are the most common cause of this neuromuscular weakness, which may affect skeletal muscles thus resulting in failure to wean from the ventilator. ICU-AW is a relevant component of PICS, with an important contribution to the residual functional disability after ICU. Thus, if it is reasonable to assume that recovery from mild COVID-19 may be accompanied by the total or near-total disappearance of fatigue and myalgia, patients with severe infection requiring mechanical ventilation may be affected by such persistent disabling manifestations, even after hospital stay.

To date, the mechanisms of the functional sequelae of critically ill patients after ICU have been investigated but not yet fully understood. In this regard, several hypotheses have been done (eg, secondary pulmonary fibrosis, organising pneumonia, chronic thromboembolic pulmonary hypertension).^3–5^ However, the need for a rehabilitative intervention both in the acute phase and after discharge seems to be a logical consequence of the currently available evidence for severe and critical patients with SARS-CoV-2 infection.

During ICU stay, when a minimum of clinical stability has been reached, patients with COVID-19 may benefit from passive mobilisation, neuromuscular electrical stimulation, frequent position changes and gradual achievement of the antigravity posture. The importance of an adequate positioning can be better understood if we consider the evidence that patients with COVID-19 may take advantage from prolonged prone position in the early acute phase. A multidisciplinary management of invasive ventilation is also required, in order to reduce the risk of ventilator-induced lung injury and to allow for a progressive ventilation weaning.

In the post-acute phase, recovery of pulmonary function, exercise capacity and quality of life after specialised pulmonary rehabilitation have been demonstrated in survivors of ARDS due to severe H1N1 influenza.^6^ However, the correct timing for exercise training and multidisciplinary rehabilitation after ICU is still to be defined for patients with COVID-19. Similarly, considering the wide range of clinical presentations of the disease, different rehabilitation strategies according to disease severity should be defined.

Whether non-severe patients may benefit from a structured rehabilitative approach is still uncertain. Some preliminary data investigating the prevalence of residual functional limitations among patients with mild COVID-19 showed high prevalence of sleep and mood disorders.^7^ However, in order to minimise the risk of contact and contagion in this period of global emergency, telemedicine and telerehabilitation may represent a valid support for non-severe patients with SARS-CoV-2 infection. In keeping with this, although unable to address the whole range of requirements of a traditional rehabilitation therapy, virtual reality and commercial exercise games (exergames) may help to maintain an adequate level of physical activity at home, especially in younger patients who are the most likely to be compliant to this type of training.^8^

In contrast, an intensive in-hospital rehabilitative approach may be required for patients with severe and critical COVID-19. SARS-CoV-2 infection seems to determine longer ventilation time and, therefore, higher levels of deconditioning than in general ICU patients. The diagnosis of critical illness myopathy and polyneuropathy is made by means of electrodiagnostic studies, and this is crucial for an adequate management of the neuromuscular weakness after ICU discharge. Moreover, several other complications (eg, post-extubation dysphagia, tracheostomy-related problems, bedsores, thromboembolism, urinary dysfunction) may affect patients with COVID-19 after the acute phase, thus needing specific rehabilitative programmes.

Moreover, current literature data suggest that SARS-CoV-2 is not confined to the respiratory tract and a multiple organ involvement seems to occur in critically ill patients. Therefore, in the attempt to predict and prevent the long-term consequences of severe SARS-CoV-2 infection, a further aspect to consider is the high incidence of cardiac and neurologic manifestations, thus calling into question the need for both prevention strategies and cardiac or post-stroke rehabilitation programmes.

Overall, the complexity and variability of COVID-19 manifestations support the hypothesis that the long-term consequences of severe SARS-CoV-2 infection cannot be considered from a single point of view, namely an acute infective disease of the respiratory tract potentially resulting in ARDS. Thus, it is reasonable to assume that the management of such patients after the acute phase cannot depend on a single generalised approach but rather on a patient-tailored multidisciplinary approach, which may be delivered by appropriate rehabilitation programmes.

In the past months, most efforts by governments and healthcare providers have been directed towards adequate treatment of patients with COVID-19 while adopting appropriate containment and mitigation strategies. As a consequence, rehabilitation services have been interrupted in some regions, while some rehabilitation facilities have been reorganised to enlarge the critical care capacity. Understanding the needs of a population is mandatory to plan an effective response in a period of global emergency. About 15% of the world’s population lives with some form of disability, with higher levels of healthcare necessity and important public health implications. The unmet need for rehabilitative treatments may be further exacerbated by the pandemic, both as a consequence of the containment strategies and of the increased demand.

A growing amount of literature data suggests that the residual functional disability after the acute phase should not be considered as a secondary or marginal aspect of the pandemic. Accordingly, the increased need for essential rehabilitation services should not be ignored. Thus, in the frame of a primary healthcare policy, while ensuring the safety of both patients and caregivers, new measures to monitor the functional outcomes of COVID-19 should be adopted, and the link between community-based rehabilitation programmes and specialised rehabilitation centres should be urgently implemented.
